# Imbalance of NET and Alpha-1-Antitrypsin in Tuberculosis Patients Is Related With Hyper Inflammation and Severe Lung Tissue Damage

**DOI:** 10.3389/fimmu.2018.03147

**Published:** 2019-01-10

**Authors:** Mayla Gabryele Miranda de Melo, Eliene Denites Duarte Mesquita, Martha M. Oliveira, Caio da Silva-Monteiro, Anna K. A. Silveira, Thiago S. Malaquias, Tatiana C. P. Dutra, Rafael M. Galliez, Afrânio L. Kritski, Elisangela C. Silva, the Rede-TB Study Group

**Affiliations:** ^1^Molecular Mycobacteriology Laboratory, Medical School-Federal University of Rio de Janeiro, Rio de Janeiro, Brazil; ^2^Ary Parreira Institute-State Secretary of Health of Rio de Janeiro, Niteroi, Brazil; ^3^Development Center for Technology on Health, CDTS-Fiocruz, Rio de Janeiro, Brazil; ^4^Tuberculosis Academic Program-Medical School-Federal University of Rio de Janeiro, Rio de Janeiro, Brazil; ^5^Laboratory of Biology Recognize, Center of Bioscience and Biotechnology, State University of North Fluminense Darcy Ribeiro, Rio de Janeiro, Brazil

**Keywords:** severe pulmonary tuberculosis, neutrophils, NET, alpha-1-antitripsin, hyperinflammation, biomarkers

## Abstract

**Background:** Pulmonary tuberculosis (PTB) can lead to lung tissue damage (LTD) and compromise the pulmonary capacity of TB patients that evolve to severe PTB. The molecular mechanisms involved in LTD during anti-tuberculous treatment (ATT) remain poorly understood.

**Methods and findings:** We evaluated the role of neutrophil extracellular trap (NET) and the occurrence of LTD through chest radiographic images, the microbial load in sputum, and inflammatory serum profile (IL-12p40/p70, IL-8, IL-17A, IL-23, VEGF-A, MMP-1, and -8, galectin-3, citrunillated histone H3—cit-H3, alpha-1-antitrypsin—α1AT, C-reactive protein—CRP and albumin) in a cohort of 82 PTB patients before and after 60 days of ATT. Using univariate analysis, LTD was associated with neutrophilia and increase of several inflammatory proteins involved in the neutrophil-mediated response, being cit-H3 the more related to the event. In the multivariate analysis, neutrophilia and cit-H3 appear as directly related to LTD. The analysis of the ROC curve at day 60 presented AUC of 0.97 (95.0% CI 0.95–1). Interestingly, at day 0 of ATT, these biomarkers demonstrated fine relation with LTD showing an AUC 0.92 (95.0% CI 0.86–0.99). Despite of that, the same molecules have no impact in culture conversion during ATT.

**Conclusions:** Our data revealed that NETs may play a key role in the pathway responsible for non-specific inflammation and tissue destruction in PTB. High level of cit-H3 and low level of α1AT was observed in the serum of severe TB patients, suggesting a breakdown in the intrinsic control of NET-driven tissue damage. These data show a new insight to knowledge TB immunopathogenesis, the role of neutrophil and NET pathway. Likewise, we identified possible biomarkers to screening of PTB patients eligible to adjuvants therapies, as anti-inflammatories and alpha-1-antitrypsin.

## Introduction

In 2016, there were an estimated 1.8 million deaths and 10.4 million new cases of tuberculosis (TB) at global level (1). World Health Organization (WHO) estimates that 480,000 subjects had multidrug-resistant TB (MDR-TB), of which 10% developed extensively drug resistant TB (XDR). In addition, pulmonary tuberculosis (PTB) may evolve toward several complications: atelectasis, bronchiectasis, secondary infections, and loss of respiratory capacity (2–5). Recently, new therapies aimed at improving treatment outcomes, shortening the duration of treatment and reducing lung pathology in TB patients have received more attention (6–8). However, few data are available regarding the lung tissue damage (LTD) and inflammatory response during anti-tuberculous treatment (ATT) (9, 10).

Non-resolving inflammation is associated with imbalance of innate immune response, neutrophilia and subsequent extensive fibrosis (11, 12). Neutrophils are the first cells to respond to an infection and participate in the adaptive immune response (13) and play a role in LTD (14–16). On the other hand, severe PTB is considered an immunopathological disease, developed due to immune hyperreactivity. TB pathology involves uncontrolled profile of pro-inflammatory cytokines and chemokines, extensive neutrophilic infiltration, exacerbated T cell responses and down-regulation of intrinsic controls mediated by Treg cells (17–21). The neutrophil acts through phagocytosis, degranulation or release of extracellular traps (NETs, neutrophil extracellular traps) (13). Such traps were first described in the last decade (22) and contain core DNA, IL-17 (23) and proteases including metalloproteinase-8 (MMP-8) (24), cathepsin-G, proteinase-3, and neutrophil elastase (NE) (13), which is a known mediator of LTD (25). In the process of NET release, core DNA histones are citrullinated to reduce its affinity for the DNA (26, 27). The immunopathogenesis induced by NETs has been described for several human diseases, infectious and non-infectious (28), being particularly important to lung diseases. A massive influx of neutrophils into the airways was found in many inflammatory lung diseases. Yet, NETs have been identified in the lungs of patients with cystic fibrosis (CF), acute lung injury, allergic asthma, and in following of infectious processes through bacteria, fungi or virus (29, 30). Platelet–neutrophil complexes are present in a wide range of inflammatory conditions including bacterial infections and sepsis (31), inflammatory bowel disease (32), and pulmonary inflammatory syndromes (15, 33). Sakurai and collaborators demonstrated that NET formation and platelet aggregation are first steps to tissue damage in murine sepsis model (34). *Mycobacterium tuberculosis* (Mtb) induces NET formation (35–37). However, neither *in vivo* nor the *in vitro*-produced NETs are able to kill the pathogen (38). MMP-8, a NET component, is elevated in sputum samples from TB patients (24). In addition, neutrophils also block the ability of resident macrophages to kill Mtb (39). Recently, Schechter et al. described that the plasma myeloperoxidase and elastase (NET component) baseline levels correlate with lung disease severity and decreased antibiotic therapy efficiency (40). However, there is no report on therapeutic approaches aimed at identifying biomarkers that regulate the deleterious effects of NETs in TB. Alpha-1-antitrypsin (α1AT) is an acute phase glycoprotein produced by the liver, but also by neutrophils, monocytes, alveolar macrophages among others (41). The role of α1AT in inhibition of neutrophil proteases is an important negative modulator of the NET activity and is considered the key function of α1AT (42). Thus, α1AT deficiency is related to lung disorders, such as emphysema and chronic obstructive pulmonary disease (COPD) due to parenchyma lesions caused by neutrophil activation (43, 44).

Galectin-3 is a β-galactosidase binding protein involved in many biological processes, such as cellular adhesion, differentiation and proliferation, apoptosis, splicing pre-RNAm and others. On the other hand, its participation has been described in several human diseases, such as cancer, fibrosis, chronic inflammation (45). Galectin-3 is an important biomarker for diagnosis and prognosis for numerous diseases and a new promising therapeutic target (45). In the lung, galectin-3 modulates the neutrophils recruitment in pneumonia and asthma. Moreover, galectin-3 is important to clearance of apoptotic neutrophils by macrophages is a key step in the resolution of inflammation in both pathological processes. In addition, galectin-3-deficient mice showed reduction in neutrophil influx and increase susceptibility to infection by *Streptococcus pnemoniae* (46). Galectin-3 is found on sputum macrophages and neutrophils in human asthma (47), although its participation in diverse asthma phenotype in not well-understood.

In summary, maintenance and amplification of the neutrophilic response, NETs pathway, may induce hyperinflammation, LTD and loss of respiratory capacity in severe PTB. Indeed, understanding host immune responses against TB may help in the development of new host-directed therapy protocols. Addressing these questions, we evaluate the inflammatory response in patients with PTB and select serum biomarkers that could predict a hyperinflammatory profile, extensive LTD and unfavorable treatment outcome.

## Methods

### Study Design

The study performed was a longitudinal cohort enrolling 82 patients bacteriologically confirmed with PTB. These patients were admitted for the TB treatment at a referral hospital in the state of Rio de Janeiro, Brazil (Instituto Estadual de Doenças do Torax Ary Parreiras), between March 2007 and December 2010. The inclusion criteria for study population were: (a) aged between 18 and 60 years; (b) both sexes; (c) positive smear microscopy -AFB or (d) positive culture for *M. tuberculosis* complex subsequently confirmed by biochemical tests. The exclusion criteria were: (a) taking anti-TB drugs before admission, (b) insulin-dependent diabetes mellitus, (c) renal failure or hemodialysis, (d) peritoneal dialysis or blood transfusion; (e) women in pregnancy or lactation period; (f) those whose clinical samples were not subjected to bacteriological or laboratory tests.

### Radiographic Evaluation

All patients underwent chest radiography in the pre-ATT and day 60 on posterior-anterior (PA) and left (PE) profile. To improve the quality of the images evaluated in this study, the radiographs were digitized at the Advanced High Performance Computing Center of the Federal University of Rio de Janeiro, Brazil. Once digitized and saved, the images were seen by two pulmonologists (EM, AK), who were blinded to clinical/demographic characteristics, laboratory results and TB treatment outcome. According to the chest X-rays of the Fleischner Society Committee (48), an standardized case report was used to document the presence of the following images: loss of volume, consolidation, nodules, enlargement of the hilar lymph nodes, pleural effusion, pleural thickening, inflammatory infiltrate, and cavities. The location of the consolidation was specified by dividing the lung into three zones (upper/mid/lower for each lung). A senior radiologist (E.T.) read each film, also blinded to the original interpretations, when disagreement occurred between two pulmonologists. Severe LTD was defined as bilateral lung disease (4 thirds or more) and cavities (at least one with more than 2 cm).

Thus, each third was analyzed for the presence of changes in pre-ATT and day 60. The classifications were categorized as: (a) improvement without or with low lung injury; (b) no improvement when lesions and lung destruction were suggestive of fibrosis and inflammation, regardless of the cure of the disease.

### Evaluation of Blood Cells and Serum Analytes

Leukocytes were counted and classified through leukogram analysis. Neutrophilia was characterized by cell count higher than or equal to 7,500 cells/mm^3^. Thrombocytosis was considered equal or above of 450,000 cells/mm^3^. C-reactive protein and albumin was quantified using nephelometry and colorimetric assay, respectively. All analyzes were performed in a certified laboratory for clinical analysis.

### Immunoassays

Luminex, model L200 (Millipore, Massachusetts, USA) was used to evaluate serum levels of, IL-8, IL-12p40, IL-12p70, VEGF-A, MMP-1, and-8 (R&D Systems, Minneapolis-USA). The results were presented in median fluorescence intensity (MFI) units.

Enzyme-linked immunosorbent assay (ELISA) was used to evaluate serum levels of citrullinated histone H3 (Cayman, Michigan-USA) and alpha-1-antitrypsin (Abcam, Cambridge-USA).

### Data Analysis

Median and interquartile scales (IQR) were used as measures of central tendency for evaluations of the population profile. Correlations between biomarkers were assessed using Pearson rank test. One-way Anova followed by multiple comparisons by Newman-Keuls was used to assess the associations of neutrophilia or radiological improvement at time 0 and 60 days of ATT with different analytes. Sputum smear microscopy and culture at 60 day of ATT was used to evaluate the efficacy of treatment among subgroups stratified from the study population. Values of *p* < 0.05 were considered statistically significant. Statistical analyzes were performed using SPSS 20.0 (IBM statistics) and Graphpad Prism 6.0 (GraphPad Software, San Diego, CA). To evaluate neutrophilia and thrombocytosis, values above 7,500 cells/mm^3^ were considered as recommended by the Brazilian society of clinical pathology.

For the multivariate analysis using logistic regression, a full imputation data set with randon forest (Rforest package in R Statistics) was built using all the variables included in the univariate analysis. The logistic regression model was built using STATA 14 and the strongest significant predictors variables were selected with a *p* < 0.05, we compared the performance of the model using the area under the receiver operator curve before the treatment and at the 60th day of treatment (49).

### Study Approval

The study was conducted in accordance with the principles set out in the Helsinki Declaration. The free and informed consent form was obtained from each participant in the study enrollment. The study was approved by the Ethics Committee of the University Hospital Clementino Fraga Filho, of the Federal University of Rio de Janeiro (protocol number: 151/05, approval number of the ethics committee: 004/05 on 04/28/2005). All information provided to the research team was encoded to maintain participant privacy.

## Results

### Study Population

Table 1A summarizes the study population sociodemographic characteristics. Of 82 patients with PTB included in the study, 87.8% were male, 63.4% were non-white, the median age was 40 years (IQR: 18–59) and the median Body Mass Index (BMI) was 17.9 kg/m^2^ (IQR: 11.8–30.7). Previous tuberculosis, HIV/AIDS, smoking, alcoholism and illicit drugs use were mentioned, respectively by 39.0, 19.5, 78.0, 70.7, and 30.5% of the participants. Only 3% of patients showed drug-resistance. 75.9% had severe tuberculosis. In baseline was observed by acid-fast bacilli (AFB) that 54.8% had low bacteriological load (1−2+), and neutrophilia was characterized in 41.5% of the patients and thrombocytosis in 62.2% (Table 1A). At pre-ATT, 53.7% (*n* = 44) of the patients showed cavitary disease and, this number increase to 63.4% (*n* = 52) after 60 days of ATT. 85.4% of the patients who presented new cavities at 60 days of ATT also presented neutrophilia (Table 1B). Interestingly, after 60 days of ATT, only TB patients with no improvement sustain neutrophilia above of 7,500 cells/mm^3^ (Table 1B) and thrombocytosis showed significant difference between radiological no improvement and improvement (Table 1B). In a multivariate analysis, all key variables, including age, gender, previous tuberculosis, life habits (chronic alcoholism, smoking history and illicit drug use) and comorbidities (HIV/AIDS; COPD) were included, and did not present as a confounder factors for this study. There was no relation of neutrophilia with other comorbidities, only with LTD.

**Table 1A:** Study population on baseline.

	**N**	**%**	***p*-value**	**OR (95%C1)**
Study population	83	100		
Male	72	86.75		
Median Age-years (IQR)	40 (18–59)			
BMI- kg/m^2^ (IQR)	17.92 (11.8–30.74)			
**ETHNIC DISTRIBUTION**				
White	31	37.35		
Non-white	5 2	62.65		
lifestyle habits				
Chronic Alcohoism (CAGE criteria)	62	74.70		
Smoking history	70	84.34		
Illicit drugs use	27	32.53		
**COMORTIDITY**				
HIV/AIDS	16	19.28		
COPD	2	2.41		
Previous tuberculosis	32	38.55		
Cavitary disease	46	55.42		
Severe Tuberculosis	63	7 5.90		
**NUMBER OF CAVITIES**				
(1–4)	29	34.94		
(5–8)	16	19.28		
≥9	1	1.20		
**POSITIVE CULTURE**^**a**^				
Favorable Outcome	32	38.55		
Unfavorable Outcome	37	44.58	0.816	0.893 (0.344–2.316)
**SMEAR GRADE**				
Negative	28	33.73		
Paucibacillary	3	3.61		
1–2+	45	54.22		
3–4+	6	7.23		
Neutrophila (≥7,500 cells/mm^3^)	47	56.63		
Favorable Outcome	15	31.91		
Unfavorable Outcome	32	68.09	0.036	0.204 (0.041–1.013)
Thrombocytosis (≥450,000 cells/mm^3^)	51	61.45		
Favorable Outcome	23	45.09		
Unfavorable Outcome	28	54.91		
Drug Resistance^b^	3	3.61		
Streptomicin; rifampicin; isoniazid	1	1.20		
rifampicin	1	1.20		
Streptomicin	1	1.20		

*Favorable Outcome or Unfavorable outcome refer to patients with improvement or no improvement radiological after 60 days of ATT*.

*IQR, interquartile range; OD, Odds Ratio; CI, confidence interval; COPD, Chronic Obstructive Pulmonary Disease; BMI, body mass index; HIV, Human Immunodeficiency Virus. ^a^Positive Culture in baseline sputum; ^b^Drug resistance to anti-tuberculosis treatment*.

**Table 1B:** Study population at 60 days of ATT.

	**N**	**%**	***p*-value**	**OR**
**CHEST-X-RAY ANALYSIS (IMPROVEMENT OR NO)**				
Yes	40	48.19		
No	43	51.80		
**Number of cavities**	55	66.26		
(1–4)	45	81.82		
(5–8)	7	12.73		
≥9	3	5.45		
New cavities	9	19.58		
New cavities + Neutrophilia	8	88.89		
**POSITIVE CULTURE**				
Favorable Outcome	4	4.81		
Unfavorable Outcome	8	9.64	0.860	0.917 (0.348–2.414)
**SMEAR GRADE**				
Negative	65	78,31		
1–2^+^	14	16.87		
3–4^+^	1	1.21		
Neutrophilia (≥7,500 cells/mm^3^)	30	36.14		
Favorable Outcome	9	30.00		
Unfavorable Outcome	21	70.00	0.001	0.174 (0.0 60–0.499)
Thrombocytosis (≥450,000 cells/mm^3^)	27	32.53		
Favorable Outcome	10	37.04		
Unfavorable Outcome	17	62.96	0.136	0.508 (0.208–1.244)

*OD, Odds Ratio; CI, confidence interval*.

### Neutrophilia Is Associated With LTD

**Figure 1:**
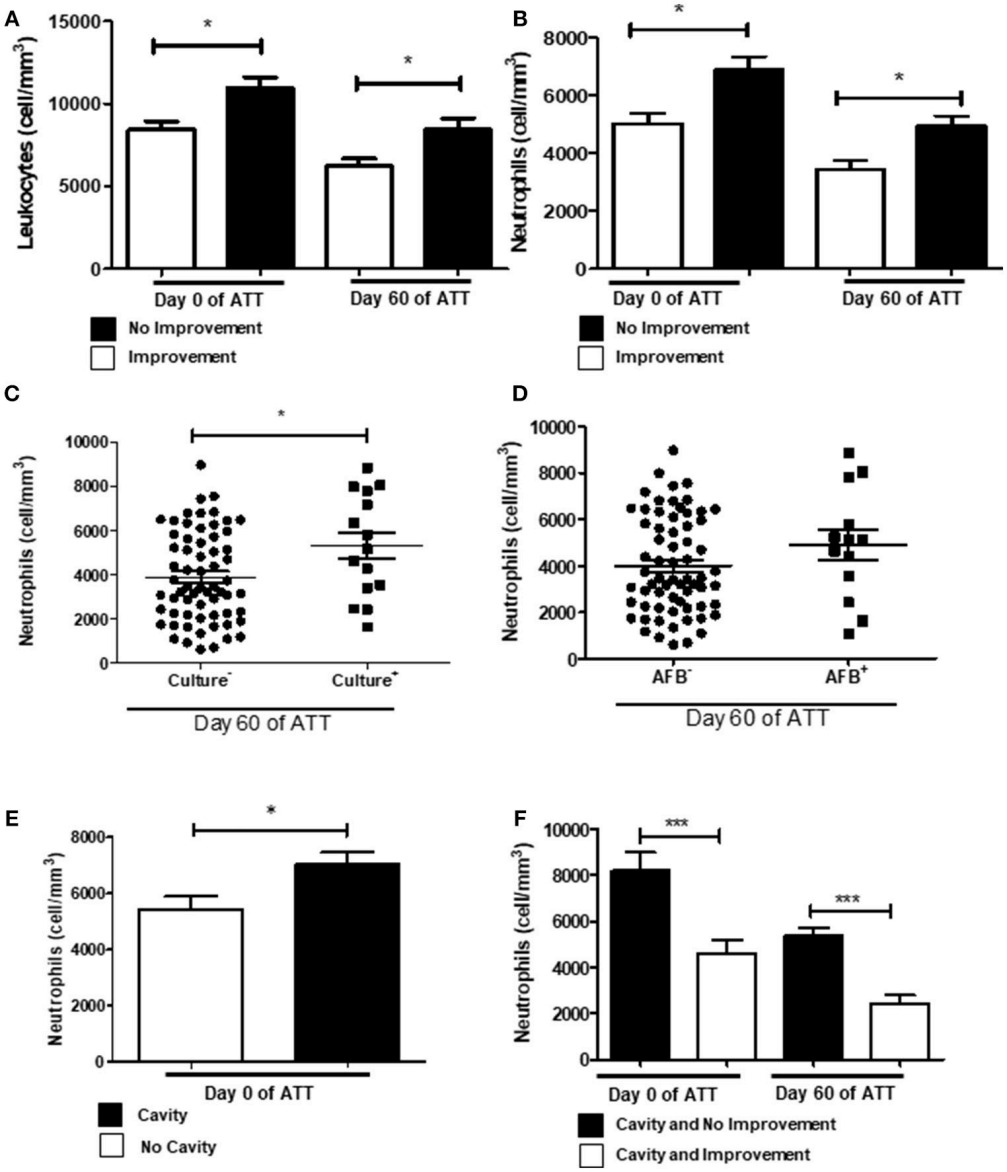
Neutrophilia and chest X-ray predict no culture conversion. **(A)** Comparison of leukocytes count between patients who presented or not radiological improvement. **(B)** Same comparison referred in “A”, with restricted analysis to the neutrophil population. **(C**,**D)** Relation of neutrophil counts and conversion of culture and AFB after 60 days of ATT, respectively **(E)** Relation of neutrophil counts and presence of cavities pre-ATT. **(F)** Influence of neutrophil counts in T0 and T60 and cavity formation and no radiological improvement after 60 days of ATT. All cell counts were expressed in cells/mm^3^. Data in each figure are expressed as mean ± SD. *p ≤ 0.05, **p ≤ 0.01 and ***p ≤ 0.0001 by 1-way ANOVA followed by Newman-Keuls test.

In our cohort, we observed that extensive LTD remained in several patients at 60 days of ATT. Figure 1A shows leukocytosis associated with LTD. To investigate which cells are involved in this process, we analyzed the patient's blood count. Figure 1B shows that neutrophils are more intensely recruited to the blood circulation of patients with LTD. Monocytes and lymphocytes were not associated with radiological improvement (Figures S1A,B). Figure 1C shows neutrophilia is significantly related to maintenance of positive culture after 60 days of ATT (*p* < 0.05). Similar results were found regarding AFB analysis, although it had not significant difference (Figure 1D). The relation to positive culture at 60 days after ATT suggests that neutrophilia may be associated with treatment failure. Indeed, neutrophilia was also associated with greater number of cavities (Figure 1E), and no improvement of radiographic images (Figure 1F).

### Acute Phase Proteins, Neutrophilia, and Thrombocytosis Are Associated in LTD

**Figure 2:**
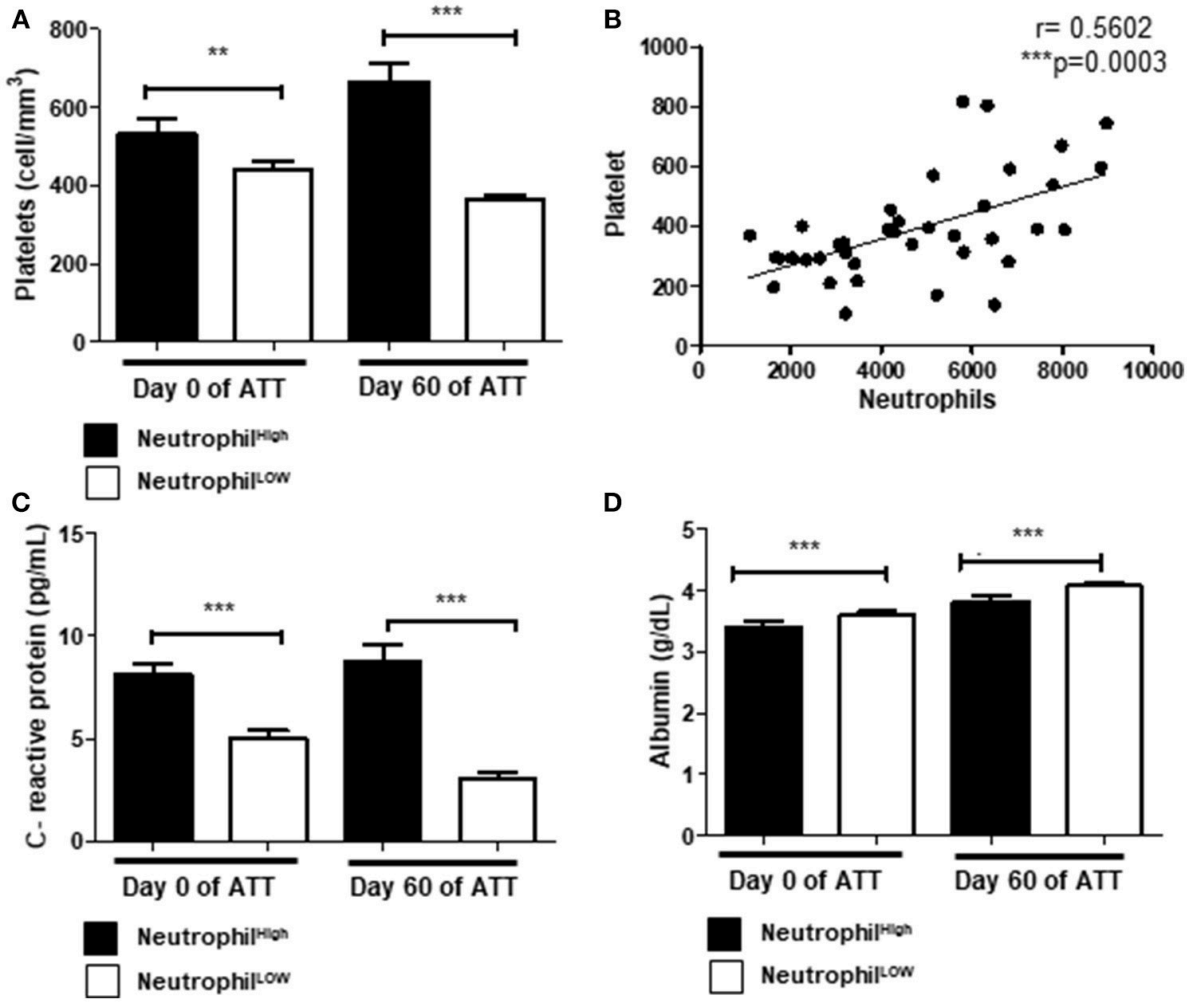
Acute phase proteins, neutrophilia and thrombocytosis are associated in LTD **(A)** Comparison of platelets and neutrophils count before and after 60 days of ATT. **(B)** Correlation of neutrophils and platelets count (*r* = 0.5602). **(C,D)** Relation of neutrophil counts and CRP or albumin, respectively. In **(A**,**C**,**D)**, “Neutrophil High” refers to neutrophilia (count ≥ 7,500 cells/mm^3^) and “Neutrophil Low” refers to normal or low neutrophils count (count < 7,500 cells/mm^3^). All cell counts were expressed in cells/mm^3^. CRP and Albumin levels were presented as pg/mL and g/dL, respectively. Data in each figure are expressed as mean ± SD. *p ≤ 0.05, **p ≤ 0.01, and ***p ≤ 0.0001 by 1-way ANOVA followed by Newman-Keuls test.

Thrombocytosis was observed in 61.5% pre-ATT and in 32.5% post-ATT (Tables 1A,B). Thrombocytosis was associated with LTD after 60 days of ATT in 62.9% of the patients (Table 1B), suggesting a role of platelets in the deleterious to anti-TB response. Additionally, neutrophilia was present in 36.1% of patients, of which 70% had an unfavorable treatment outcome (Table 1B). Indeed, neutrophils and platelets were higher in these patients, both at day 0 and 60 of ATT (Figure 2A), and significant correlation between thrombocytosis and neutrophilia was observed (*p* = 0.0003) (Figure 2B).

To investigate the role of danger signals in the neutrophilic response, we evaluated the relationship between neutrophilia and C-reactive protein (CRP) or albumin. Our results demonstrated that neutrophilia was associated with high levels of CRP (Figure 2C) and lower levels of albumin (Figure 2D). The inflammatory response observed in these patients may modulate a bone marrow overreaction, leading to neutrophilia and thrombocytosis.

### Neutrophil-Related Inflammatory Proteins Are Modulated in Severe PTB

**Figure 3:**
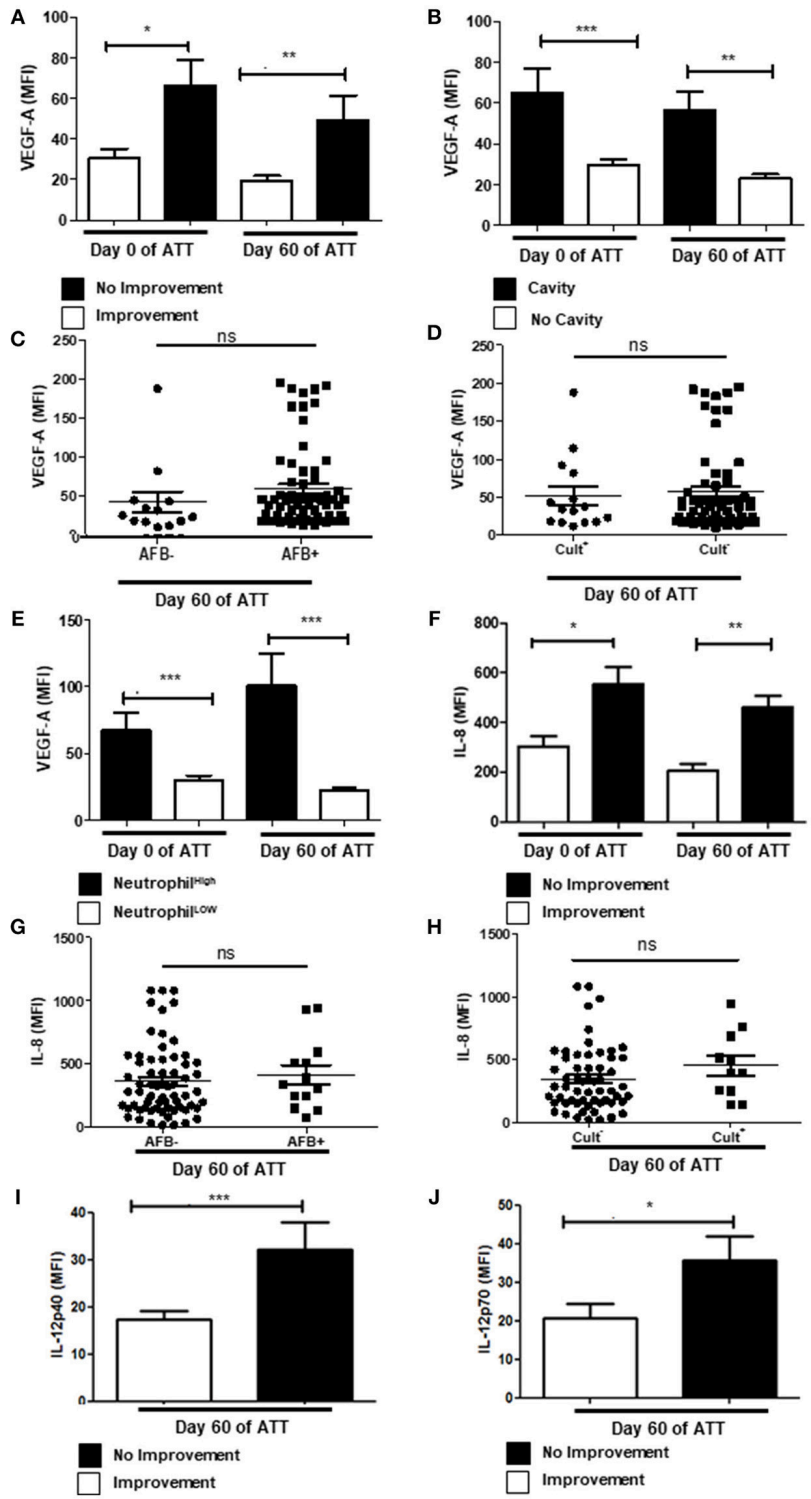
Neutrophil-related inflammatory proteins are modulated in severe PTB **(A)** Relation between VEGF-A levels in radiological improvement before and after 60 days of ATT. **(B)** Same comparison referred in “A”, with restricted analysis to cavity formation. **(C,D)** Relation of VEGF-A levels and conversion of AFB and culture after 60 days of ATT, respectively **(E)** Relation of VEGF-A and neutrophil counts. **(F)** Comparison of IL-8 levels before and after 60 days of ATT in relation to radiological improvement. **(G,H)** Relation of IL-8 levels and conversion of AFB and culture after 60 days of ATT, respectively. **(I**,**J)** Comparison of IL12p40 and IL12p70 levels between patients who presented radiological improvement or not after 60 days of ATT, respectively. All cell counts were expressed in cells/mm^3^. “Neutrophil High” refers to neutrophilia (count ≥ 7,500 cells/mm^3^) and “Neutrophil Low” refers to normal or low neutrophils count (count < 7,500 cells/mm^3^). VEGF-A, IL-8 and IL12 levels are presented as mean fluorescence intensity (MFI). Data in each figure are expressed as mean ± SD. *p ≤ 0.05, **p ≤ 0.01, and ***p ≤ 0.0001 by 1-way ANOVA followed by Newman-Keuls test.

Vascular Endothelial Growth Factor A (VEGF-A) is an angiogenic factor that contributes to granuloma vascularization and, consequently, to cell recruiting and bacteria proliferation (50–52). It has also been positively related to severity of inflammatory diseases (53), including PTB (54). In our study, we demonstrated that serum VEGF-A remained higher after 60 days of ATT in patients that presented cavities and no radiological improvement (Figures 3A,B). Regardless of those results, VEGF-A has no impact in bacteria clearance, since there is no correlation of its concentrations with AFB and culture conversion (Figures 3C,D). As VEGF-A is recognized as a chemotactic factor for neutrophils (53, 55, 56), and is produced by these cells (57, 58), we evaluated the correlation between them. Notably, we observed a positive correlation of VEGF-A and neutrophilia before and after 60 days of ATT (Figure 3E), although more remarkable after TB treatment. These results suggest a crossed enhancement of VEGF-A and neutrophils in patients with no radiological improvement at 60 days of ATT.

Interleukin 8 (IL-8) is an important pro-inflammatory mediator and induces chemotaxis, activation and NET release in human neutrophils (59). Patients who presented no radiological improvement after 60 days of ATT had higher serum level of IL-8 (Figure 3F). In addition, increased IL-8 did not related with culture conversion or negative AFB (Figures 3G,H). In addition, IL-6 was not related with LTD in PTB (Figure S2A).

IL-12 is also a major pro-inflammatory factor that positively modulates T_H_1 immune response and induces Interferon-gamma (IFN-γ) production by different cell types (60, 61). Moreover, IL-12 production is induced by Mtb infection (60, 62). Figures 3I,J show, respectively, higher concentrations of IL-12p40 and IL-12p70 in serum samples of day 60 of ATT in patients who lacked chest radiographic improvement. However, IFN-γ presented no relation with LTD (Figure S2B).

### Metalloproteinases Are Important Pieces to Enlighten Tissue Damage Mechanism

**Figure 4:**
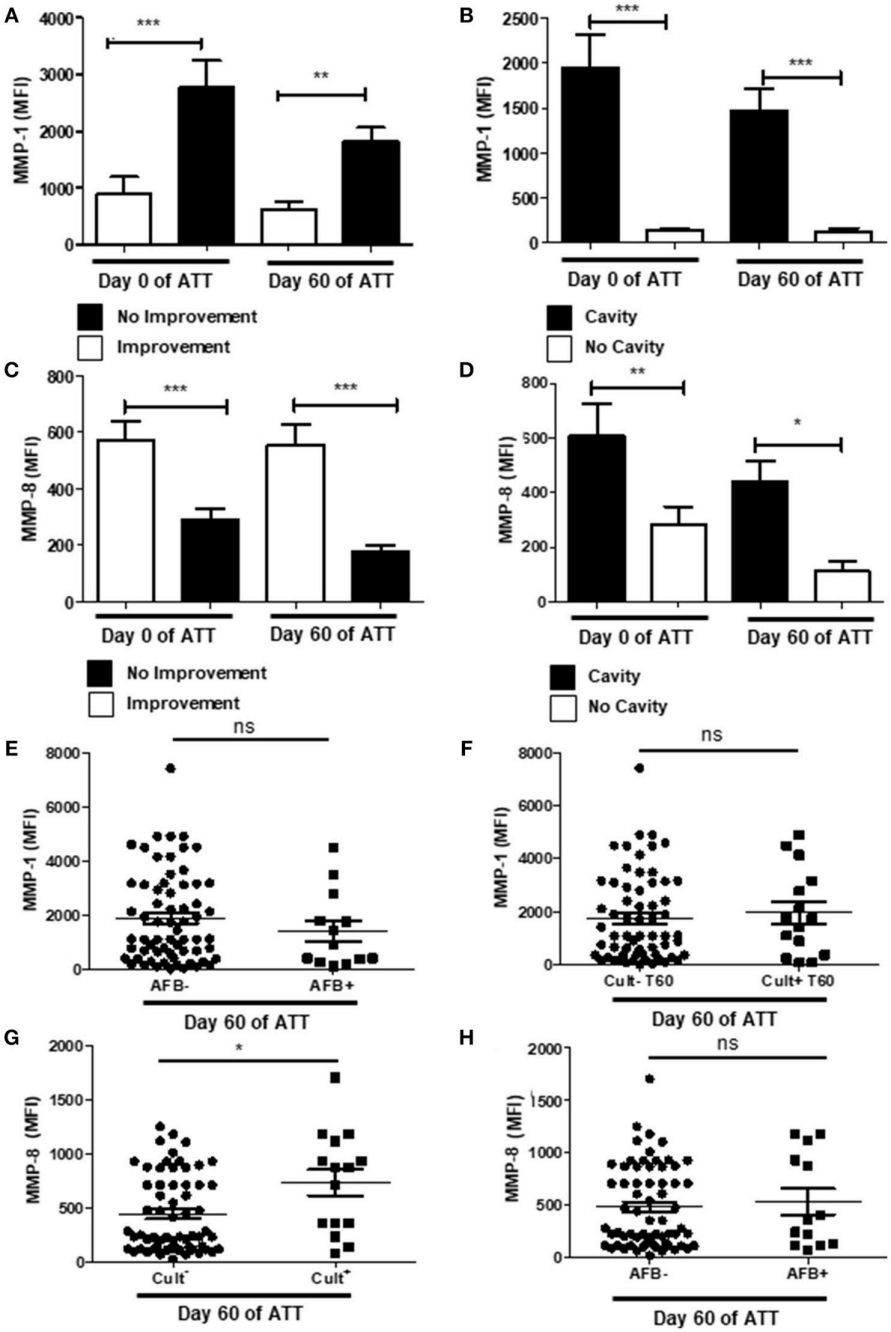
Metalloproteinases are important pieces to enlighten tissue damage mechanism**. (A**,**C)** Comparison of MMP-1 and -8 levels before and after 60 days of ATT between patients who presented radiological improvement or not after 60 days of ATT, respectively. **(B**,**D)** Same comparison referred in “A” and “C”, respectively, with restricted analysis to cavity formation. **(E**,**F)** Relation of MMP-1 levels with AFB and culture conversion, respectively. **(G**,**H)** Relation of MMP-8 levels with culture and AFB conversion, respectively. MMP-1 and MMP-8 levels are presented as mean fluorescence intensity (MFI). Data in each figure are expressed as mean ± SD. *p ≤ 0.05, **p ≤ 0.01, and ***p ≤ 0.0001 by 1-way ANOVA followed by Newman-Keuls test.

Metalloproteinases (MMPs) are zinc-dependent proteases and play important roles in cell migration, modulation of inflammatory processes and tissue damage (63). We analyzed serum concentrations of MMP-1 and -8 and its association with chest radiographic images and culture conversion at 60 days of ATT. Patients with chest-X-ray improvement presented significantly lower amounts of serum MMP-1, both before and after treatment (Figure 4A) as well as MMP-1 was increased in patients that showed cavitation in chest-X-ray images (Figure 4B). Lower serum levels of MMP-8 were associated to no radiological improvement after 60 days of treatment (Figure 4C), but patients with cavities showed higher levels of MMP-8 on day 0 and at 60 days of ATT (Figure 4D). Interestingly, MMP-1 and -8 presented inverse relation to unfavorable radiological outcome, suggesting intrinsic mechanisms modulate differentially MMP-1 and -8 production in PTB. MMP-1 and MMP-8 were also evaluated in relation to smear grade and culture. MMP-1 was not related with culture or AFB conversion (Figures 4E,F), as observed to the other mediators described above. On the other hand, MMP-8 was associated with culture conversion (Figure 4G) but not with AFB (Figure 4H).

### Galectin-3 Is Modulated in Severe PTB

**Figure 5:**
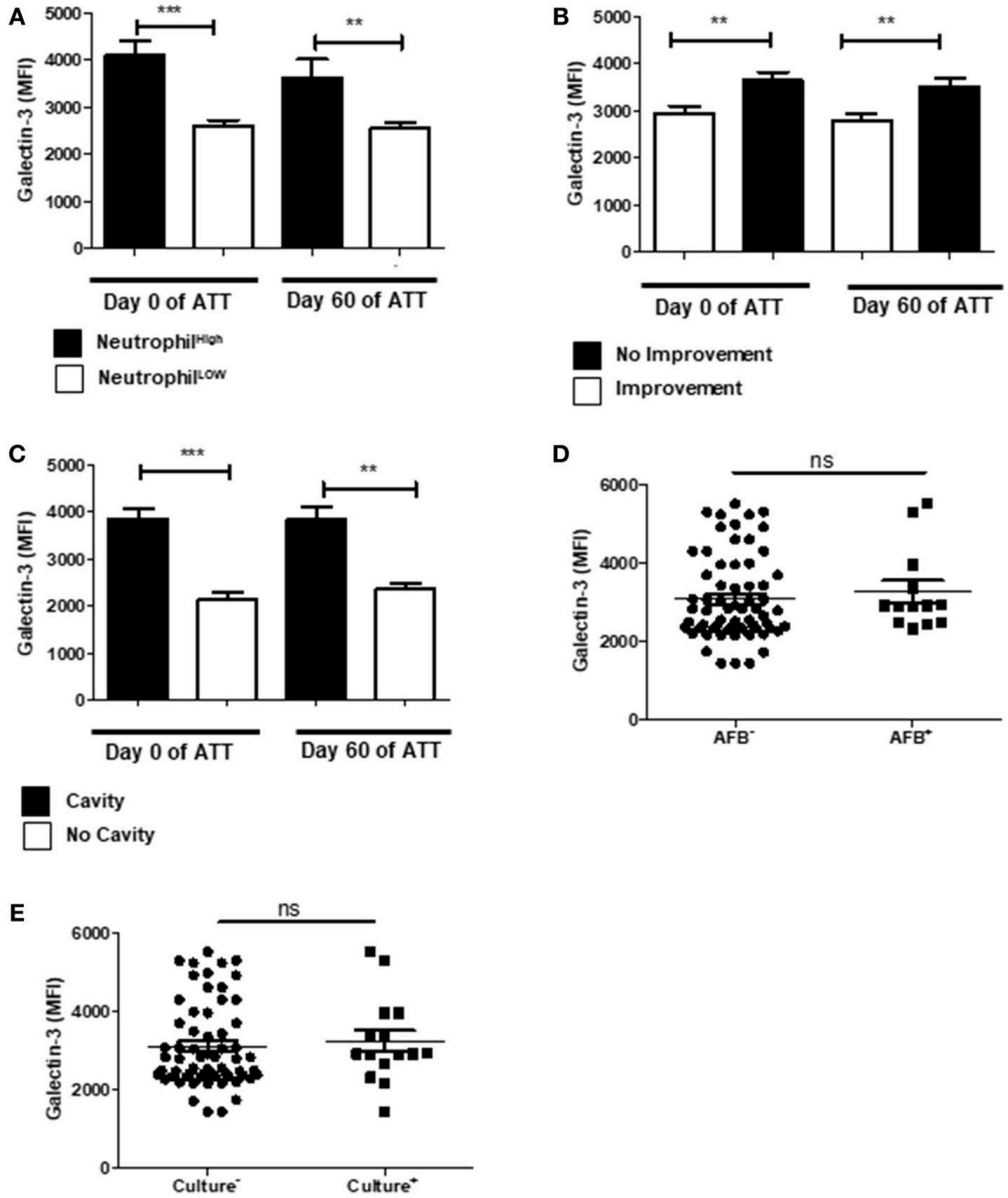
Galectin-3 is modulated in severe PTB**. (A)** Relation of Galectin-3 levels and neutrophil counts. “Neutrophil High” refers to neutrophilia (count ≥ 7,500 cells/mm^3^) and “Neutrophil Low” refers to normal or low neutrophils count (count < 7,500 cells/mm^3^). **(B)** Comparison of Galectin-3 levels before and after 60 days of ATT between patients who presented radiological improvement or not after 60 days of ATT. **(C)** Same comparison referred in “B”, with restricted analysis to cavity formation. **(D**,**E)** Relation of Galectin-3 levels and conversion of AFB and culture after 60 days of ATT, respectively. Galectin-3 levels are presented as mean fluorescence intensity (MFI). Data in each figure are expressed as mean ± SD. *p ≤ 0.05, **p ≤ 0.01, and ***p ≤ 0.0001 by 1-way ANOVA followed by Newman-Keuls test.

Galectin-3 is a β-galactosidase-recognizing protein and is an important neutrophil response modulator, inducing chemotaxis of these cells and LTD in neutrophil-mediated COPD (64). In our cohort, increased galectin-3 was associated to neutrophilia (Figure 5A). In addition, galectin-3 was higher in patients with LTD and cavity (Figures 5B,C), but was not associated with mycobacteria clearance (Figures 5D,E).

### IL-17 Downregulation Is Related With LTD

**Figure 6:**
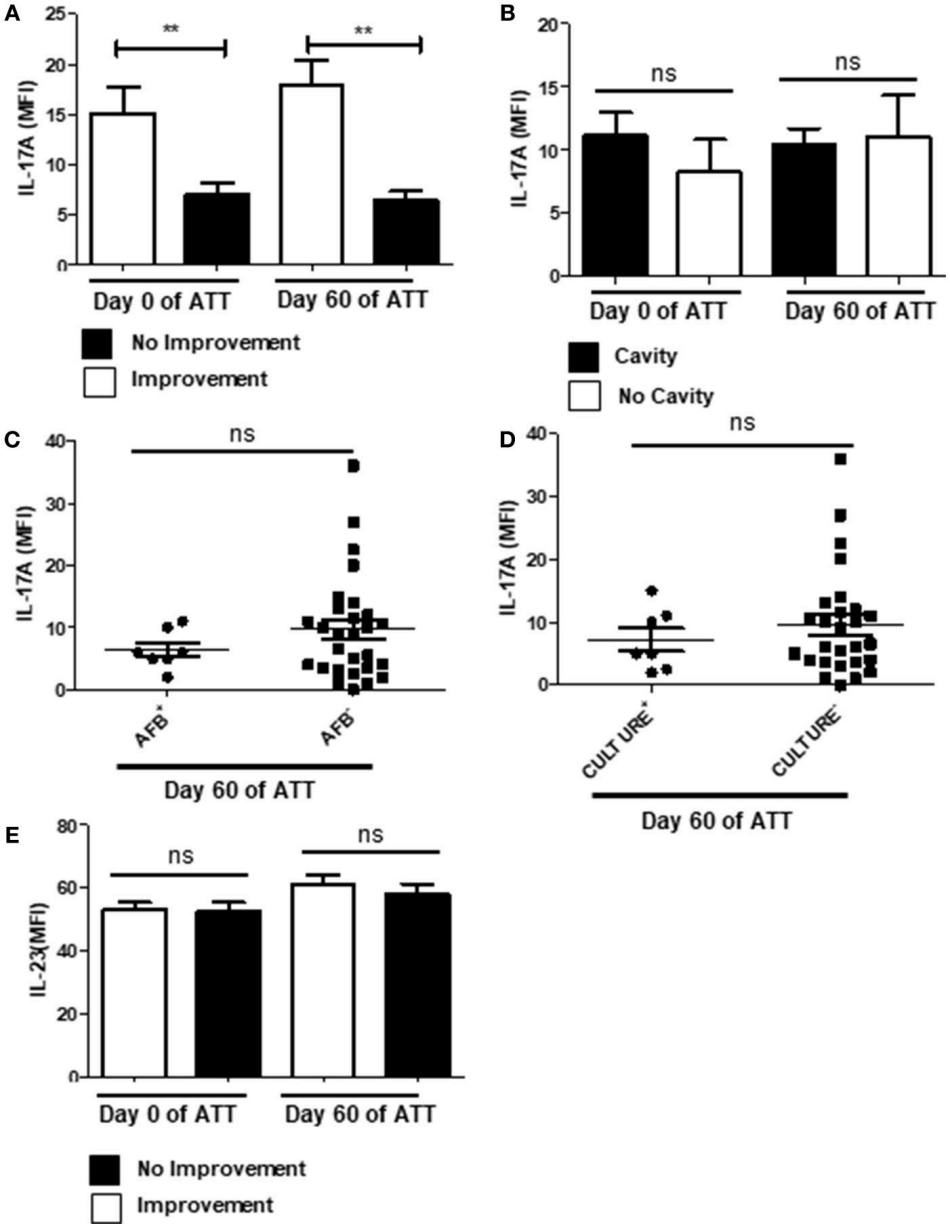
PTB showed IL-17 downregulation related with LTD no improvement. **(A)** Comparison of IL-17 levels before and after 60 days of ATT between patients who presented radiological improvement or not after 60 days of ATT. **(B)** Comparison of IL-17 levels in patients who presented or not cavity formation. **(C**,**D)** Relation of IL-17 levels with AFB and culture conversion, respectively. **(E)** Serum IL-23 IL-17 levels are presented as mean fluorescence intensity (MFI). Data in each figure are expressed as mean ± SD. **p ≤ 0.01 by 1-way ANOVA followed by Newman-Keuls test.

IL-17 is involved in neutrophil recruitment and activation through induction of IL-8 and IL-6 release (65, 66), in addition is expected to be found within the NETs (23). Thus, we investigated IL-17 levels and observed that patients who presented radiological improvement after 60 days of ATT presented significantly higher concentrations of IL-17 in the bloodstream (Figure 6A), which could suggest a protective role. However, we did not observe the same pattern in a cavity-focused analysis (Figure 6B) and IL-17 had no role in AFB and culture conversion (Figures 6C,D). Still, IL-23 was not modulated during ATT (Figure 6E), suggesting that regulatory mechanism of IL-17 secretion was preserved.

### NET Is the Main Pathway to LTD in Severe PTB

**Figure 7:**
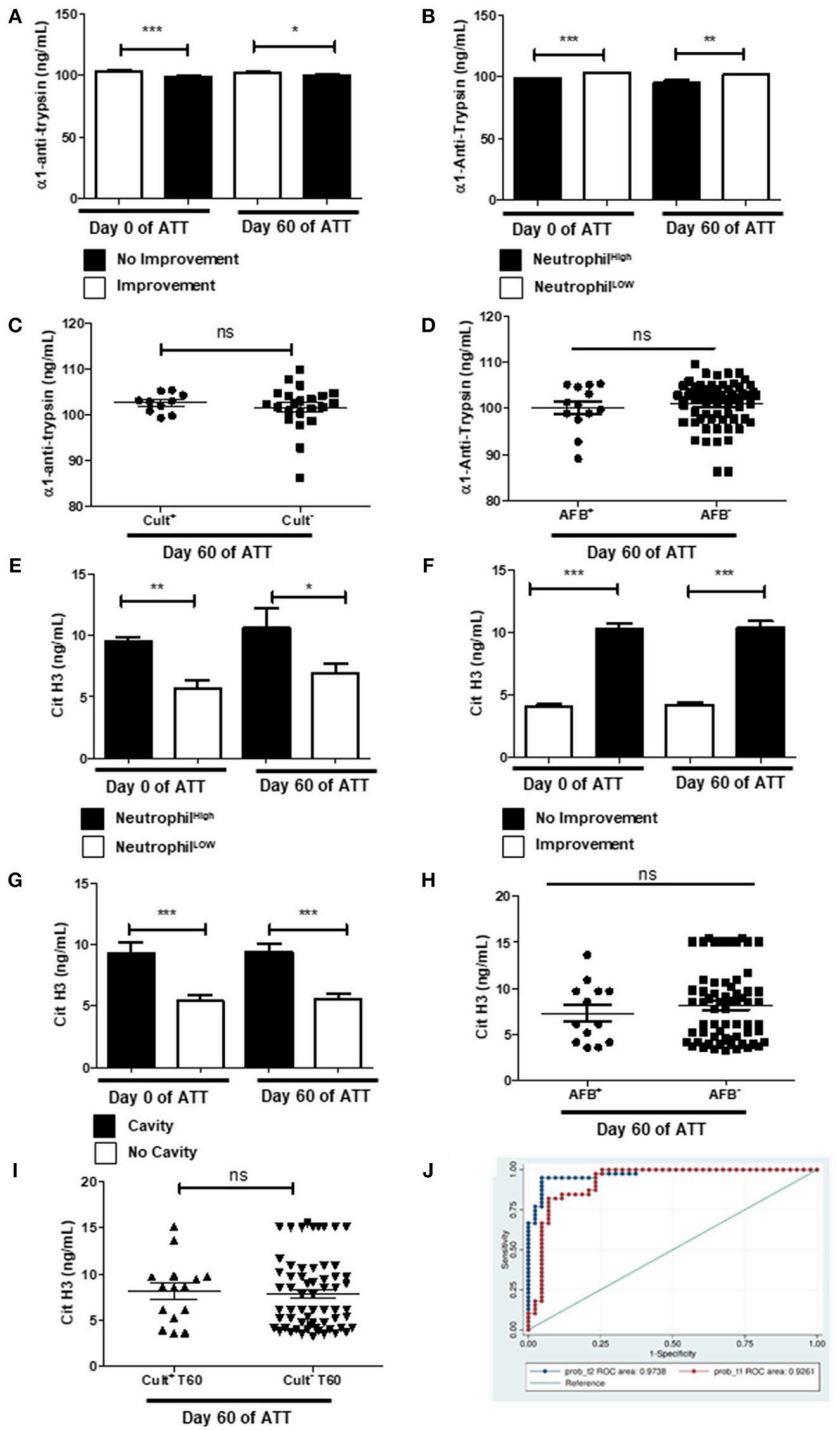
NET is the main pathway to LTD in severe PTB **(A)** Evaluation of alpha-1-antitrypsin (α1AT) levels before and after 60 days of ATT inpatients with radiological improvement or not after 60 days of ATT. **(B)** Relation of α1AT levels and neutrophil counts. **(C**,**D)** Relation of α1AT levels with culture and AFB conversion, respectively. **(E)** Relation of citrullinated histone H3 (cit-H3) levels and neutrophil counts. **(F)** Comparison of cit-H3 levels before and after 60 days of ATT between patients who presented radiological improvement or not after 60 days of ATT. **(G)** Comparison of cit-H3 levels in patients who presented or not cavity formation. **(H**,**I)** Relation of α1AT levels with AFB and culture conversion, respectively. In graphs “B” and “E”, “Neutrophil High” refers to neutrophilia (count ≥ 7,500 cells/mm^3^) and “Neutrophil Low” refers to normal or low neutrophils count (count < 7,500 cells/mm^3^). α1AT and cit-H3 levels are presented as ng/mL. Data in each figure are expressed as mean ± SD. *p ≤ 0.05, **p ≤ 0.01 and ***p ≤ 0.0001 by 1-way ANOVA followed by Newman-Keuls test. **(J)** Overlay of ROC curve at day 0 and 60 of ATT, revealed that only neutrophil, cit-H3 and α1AT day showing are related with LTD in PTB. The logistic regression model was built using STATA 14 and the strongest significant predictors variables were selected with a *p* < 0.05.

Alpha-1-antitrypsin (α1AT) is a well-studied serine protease inhibitor and prevents autoproteolytic damage (67). Before and after 60 days of ATT, α1AT levels were higher in patients who presented chest radiographic improvement after 60 days of ATT (Figure 7A) and it was downregulated in patients with neutrophilia (Figure 7B). However, α1AT was not associated with culture and AFB conversion (Figures 7C,D). Since α1AT controls neutrophil-driven LTD, these results suggest an imbalance in that protective response might allow tissue damage in PTB.

Cit-H3, on the other hand, has been proposed to be used as a marker of NET formation (68) and thus, as a marker of LTD as well. To confirm our hypothesis that NET formation is the main neutrophils' means of action responsible by LTD in our cohort, we measured cit-H3 levels in patients' serum. Figure 7E shows that patients with neutrophilia had significantly higher levels of serum cit-H3at day 60 of ATT. It was observed that patients with no radiological improvement had high titers of cit-H3 in pre-ATT and after 60 days of ATT (Figure 7F). Remarkably, patients with higher cit-H3 levels also exhibited increased cavity formation (Figure 7G), but cit-H3 was not related to negative AFB and culture conversion after ATT (Figures 7H,I). The multivariate model found Cit-H3 and neutrophils as positive markers of LTD and α1AT as a negative marker, the AUC of the model at 60th day of treatment was 0.97 (95.0% CI 0.95–1) and before the treatment it was 0.92 (95.0% CI 0.86–0.99) (Figure 7J). This result suggests that the NET pathway may be related with the LTD in TB patients and, thus, can play a central role in the pathophysiology of this process.

## Discussion

In the present study, we suggest cellular and molecular mechanisms linked to neutrophil response during ATT induce LTD in severe PTB. We observed that patients with extensive LTD presented higher neutrophil count pre- and post-ATT. Additionally, neutrophilia does not seem to favor the patient in the battle against *M. tuberculosis*, as patients with neutrophilia remain with positive AFB and culture after 60 days of ATT, confirming previous literature data (38).

NETs released as a host defense mechanism may be associated with LTD, due to imbalance of innate immune response (16, 30, 69). We demonstrated that NETs may be important mechanism inductor of LTD among PTB patients. It was clear that patients with marked LTD and neutrophilia have higher levels of cit-H3 in peripheral blood and low levels of α1AT. Cit-H3 had no relation with bacterial load, as culture conversion at day 60 of ATT was independent of cit-H3 titers. Together, these data reinforce our hypothesis that NETs may be responsible for LTD as result of an autoinflammatory process due to a breakdown in control neutrophil response, even though they are not able to clear *M. tuberculosis*. Indeed, a recent review discuss the incapacity of neutrophils in killing *M. tuberculosis* in several *in vitro* studies (38). Beyond that, the study performed in murine model suggests that NETs may provide a platform for extracellular Mtb growth and therefore contribute to the rapid enlargement of the pulmonary lesions (70). Interestingly, oral administration of heat-killed *Mycobacterium manresensis* prevents excessive neutrophilic infiltration of the lesions and preventing severe PTB (71).

It has been described that 1.5–3.4% of TB patients may develop deep venous thrombosis (72). Activated platelets induce amplification of several neutrophilic responses including phagocytosis, reactive oxygen species (ROS) production and NETs, and act in the TB pathology (68, 73). In our study, we observed that patients with tuberculosis presented thrombocytosis, which was related to LTD and neutrophilia. Those results suggest a possible interaction between neutrophils and platelets that might be responsible for an amplification loop of neutrophil-mediated autoinflammatory response and non-specific tissue damage in severe PTB.

CRP and albumin are liver-produced proteins that acts as homeostasis breakers and thus, could be used as biomarkers. In our cohort, CRP is increased in unfavorable outcome patients, confirming previous results of the group (10). Conversely, albumin decreases in these patients, on day 0 and 60 of ATT, when compared with patients with favorable treatment outcome (10). In addition, CRP and albumin show a fine relation with neutrophilia and unfavorable treatment outcome. Our results suggest that the acute phase proteins may induce neutrophilia and thrombocytosis, acting as danger signals to bone marrow and stimulating hematopoiesis. We also observed higher VEGF-A levels among patients with no radiological improvement, persistence of cavity at day 60, and a positive correlation of VEGF-A serum concentration and neutrophilia in TB patients. Those results confirm the association of VEGF-A and severity of inflammatory pulmonary TB as described by Kumar et al. (54).

Pro-inflammatory cytokines, IL-8 and IL-12, involved with neutrophilic response, appeared in higher levels among patients with LTD. IL-8 induces NET release in human neutrophils (59) and has been associated with autoinflammatory profile in inflammatory bowel diseases and COPD (74, 75), with autoimmune diseases mediated by neutrophils (76) and with tuberculosis pathogenesis (77, 78). We also evaluated the role of galectin-3 and metalloproteinases (MMP-1 and 8) in the LTD as they may be associated with neutrophil activation (79). In our cohort, galectin-3 appears as a biomarker for LTD and cavity in patients with tuberculosis during initial phase of ATT. Our data revealed that both MMP-1 and MMP-8 are increased in patients with cavity, but only MMP-1, which is a chemotactic factor for neutrophils and modulates the influx of these cells (80), is elevated in patients with no radiological improvement, consistent with previous observations (79). Besides, MMP-8 serum level was enhanced in patients with positive culture after 60 days of ATT. Regarding the MMP-8 results, its inverse relation to LTD was interestingly unexpected, as it is a component of NETs (24) and in the light of our results already described. Previous studies detected MMP-8 available in pulmonary secretions (sputum, bronchoalveolar lavage) from PTB patients, as well as confirmed the presence of neutrophils expressing MMP-8 in the cavity wall (reviewed in the reference 81). According to the pathophysiology described in the literature, Mtb triggers the production of MMP-1 and initiates the early destruction of inflammatory foci, leading to subsequent cavity maturation by neutrophils pre-stored MMP-8 [reviewed in (81)]. Therefore, we suggest that our results might be a consequence of MMP-8 retention in the lung parenchyma, which limits its detection in the serum.

IL-17 is essential for establishment of early granuloma and protective T_H_17 response during TB infection (65, 82). Our data show that lower concentrations of serum IL-17 are related with unfavorable outcome and LTD. IL-17 is produced by T_H_17 cells, neutrophils and others cells. The IL-17 production by T cells is supported by IL-22, TGF-β, IL-6, TNF-α, and IL-1. Interestingly, none of these cytokines was modulated in our study population. Furthermore, IL-23, which is essential to maintenance of IL-17 production by T_H_-17 cells (82, 83), was not negative- or positively modulated (84). All our results suggest that axis IL-23/IL-17 related with T_H_-17 cells was not modulated in these patients, suggesting that the decrease in serum IL-17 may be related with neutrophilic response and NETs, modulated in our cohort. Conversely, our results show serum IL-23 was not differentially modulated in patients with favorable and unfavorable TB treatment outcome, suggesting that response mediated by T_H_17 cells was not affected and, therefore, that the differential production of IL-17 is derived from another axis. Recently, a review about neutrophils and severe TB, describes the PI-3K role in neutrophils recruitment to the lung and LTD (84). As highlighted in the review, PI-3K modulates the IL-17 expression in the lung parenchyma and neutrophil influx, acting on the IL-17/G-CSF axis. The authors described that increase or decrease of expression of PI-3K to favor together susceptibility or severity to tuberculosis, respectively, both in murine model and humans (84). Additionally, Mtb modulates PI-3K expression by pulmonary parenchyma cells, such as epithelial cells, favoring influx of neutrophils and hyperresponsiveness and LTD, independently of T_H_17 cells role, as proposed by Leisching (84). As neutrophils produce IL-17 and release it through NETs (85), we suggest IL-17 is probably being sequestrated by pulmonary parenchyma, as it might be happening to MMP-8. That would be consistent with previous observations showing that overexpression of IL-17 is related to the severity of asthma disease (66) and as has been described in murine model, the increase of intrapulmonary IL-17 is related with LTD (86). Cruz et al. also suggest that the imbalance in IL-17 response does not interfere in the Mtb clearance, which is consistent with our results (86). Thus, we suggest that the immune response profile generated during initial phase of infection may direct the immune response to hyperinflammatory (neutrophil-mediated) or inflammatory (macrophage-mediated). This dichotomy can predict the outcome of treatment and the profile of damage tissue.

**Figure 8:**
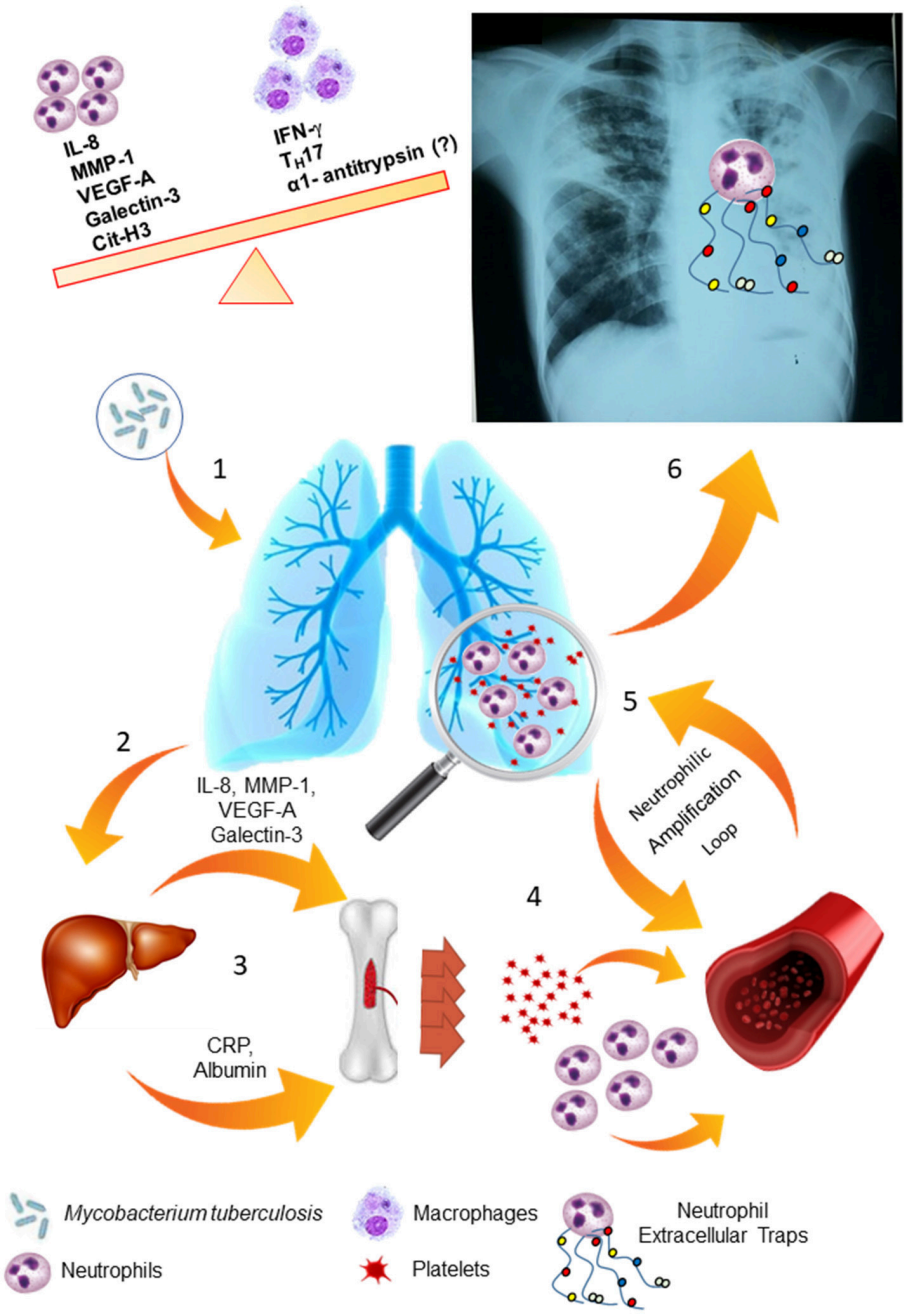
LTD is based on the maintenance of the neutrophilic response and its amplification loops. (1) *Mycobacterium tuberculosis* infects the lung tissue and generates an inflammatory response through primary infection or reactivation of latent TB infection. (2) Several soluble molecules, such as VEGF-A, MMP-1, Galectin-3 and IL-8 are released in the site of infection and modulate the liver. (3) Liver-produced acute phase proteins (CRP and albumin) and soluble molecules from the infection site modulate the bone marrow. (4) In response to these stimuli, the bone marrow mobilize neutrophils and platelets to the blood flow and enhance the production of these cells. (5) These cells are attracted to the lung tissue, where they are activated and produce larger amounts of VEGF-A, IL-8 and MMP-1, maintaining the response in an amplification loop. (6) Neutrophils are activated by platelets and release Neutrophils Extracellular Traps (NETs), which contains MMP-8 and elastase, and lead to lung tissue damage.

Indeed, our results suggest an imbalance of neutrophilic response may result in LTD and impaired therapy. Figure 8 summarizes our proposal to the biological response involved in severe PTB. A strong activation of the bone marrow mediated by acute-phase proteins and cytokines may lead to neutrophilia and thrombocytosis, as well as chemotactic and inflammatory proteins that participate of neutrophil response are increased in serum of severe PTB and are associated with persistence of tissue damage, as seen in chest-X-ray images. Thus, all events would culminate in a strong and neutrophil-mediated autoinflammatory response and subsequent LTD in severe PTB.

Unfortunately, it is worth pointing out that as our study was based exclusively on serum analysis, it did not allow a specific evaluation of the ongoing processes within the pulmonary microenvironment. Thus, it cannot be confirmed that the serum inflammatory mediators evaluated in this study can be modulated in the pulmonary parenchyma, despite of the strong relationship with LTD diagnosed by chest X-ray. Evidently, further histopathological studies are necessary to describe and confirm our hypothesis, especially the MMP-8 and IL-17 sequestration in the lung parenchyma. Peripheral blood mononuclear cells were not collected for neutrophil and T cell phenotype evaluation in this cohort. On the other hand, the low drug resistance rate and the enrollment of only inpatients allow us to put aside the drug resistance and the poor treatment adherence as contributing factors to lung damage, severe PTB and treatment failure.

The several proteins involved in the neutrophilic response are elevated in patients with severe LTD. Serum cit-H3, may be used as a potential biomarker suitable to predict unfavorable outcome in PTB before treatment, eligible to host-directed therapies concerning adjuvant anti-inflammatory drugs, considering its profile as screening test to LTD. Neutrophilic response does not contribute effectively to mycobacteria clearance. Thus, host-directed therapies aimed at down regulating this autoinflammatory response might control LTD without impairment of antibiotic treatment. α1AT may be potential candidate to adjuvant therapy in hyperinflammatory patients. In addition, revealing biomarkers available in serum implies a non-invasive sampling approach. The biomarkers evaluated in this paper are easily measured through economical and ordinary methods that do not require highly trained professionals or high-technology equipment.

## Author Contributions

MdM and ES designed and performed experiments and wrote the manuscript. EM and AK evaluated the chest-X-ray images. MO designed and supervised the database and biorepository used in this project. ES performed the univariate statistical analysis and RG, the multivariate ones. TD, TM, CS-M, and AS provided help in performing experiments. ES and AK supervised the project and edited the manuscript.

### Conflict of Interest Statement

The authors declare that the research was conducted in the absence of any commercial or financial relationships that could be construed as a potential conflict of interest. The handling Editor declared a shared affiliation, though no other collaboration, with several of the authors MdM, MO, CS-M, AS, TM, TD, RG, AK, ES.

## Supplementary Material

Figure S1 Monocytes and lymphocytes are not expressive in LTD process. **(A)** Comparison of monocytes count between patients who presented or not radiological improvement. **(B)** Comparison of lymphocytes count between patients who presented or not radiological improvement. All cell counts were expressed in cells/mm^3^. Data in each figure are expressed as mean ± SD. ns–p, non-significant by 1-way ANOVA followed by Newman-Keuls test.

Figure S2 IL-6 and IFN-γ are not related with radiological improvement. **(A)** Comparison of IL-6 levels before and after 60 days of ATT between patients who presented radiological improvement or not after 60 days of ATT. **(B)** Comparison of IFN-γ levels in patients who presented or not cavity formation. Serum IL-6 and IFN-γ levels are presented as mean fluorescence intensity (MFI). Data in each figure are expressed as mean ± SD. ns–p, non-significant by 1-way ANOVA followed by Newman-Keuls test.
