# Supplementary figures and images for: Imbalance of NET and Alpha-1-Antitrypsin in Tuberculosis Patients Is Related With Hyper Inflammation and Severe Lung Tissue Damage
Source: Front Immunol. 2019 Jan 10;9:3147. doi: 10.3389/fimmu.2018.03147 (PMC6335334; doi:10.3389/fimmu.2018.03147)

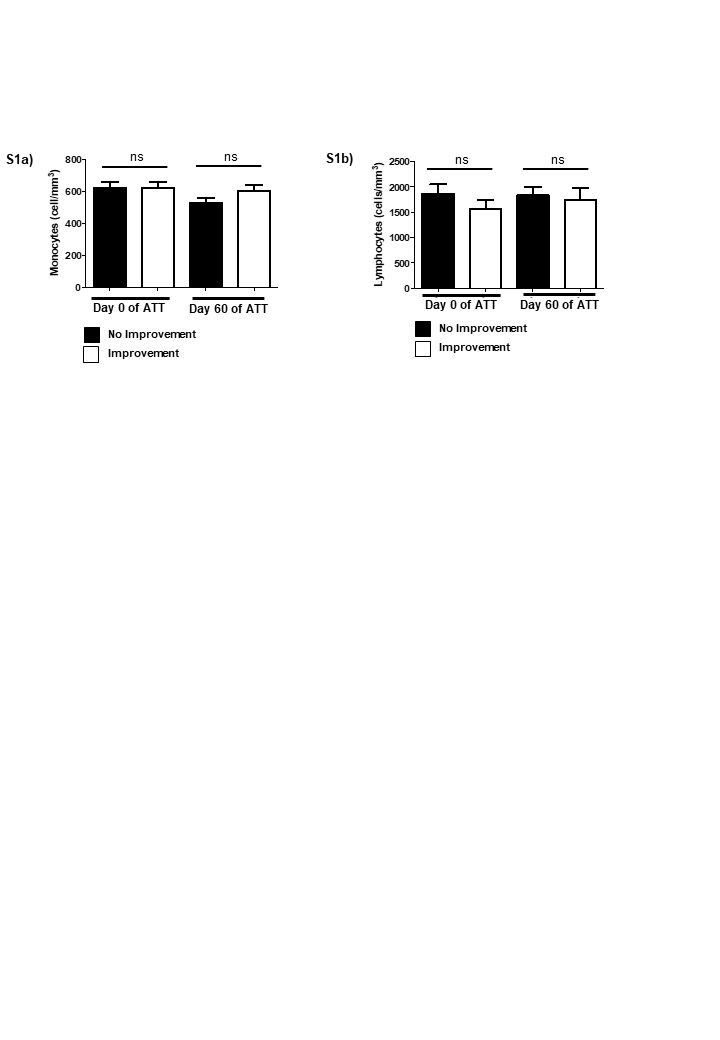

Supplement: Figure S1 — Monocytes and lymphocytes are not expressive in LTD process. (A) Comparison of monocytes count between patients who presented or not radiological improvement. (B) Comparison of lymphocytes count between patients who presented or not radiological improvement. All cell counts were expressed in cells/mm3. Data in each figure are expressed as mean ± SD. ns–p, non-significant by 1-way ANOVA followed by Newman-Keuls test. [file Image_1.TIF]

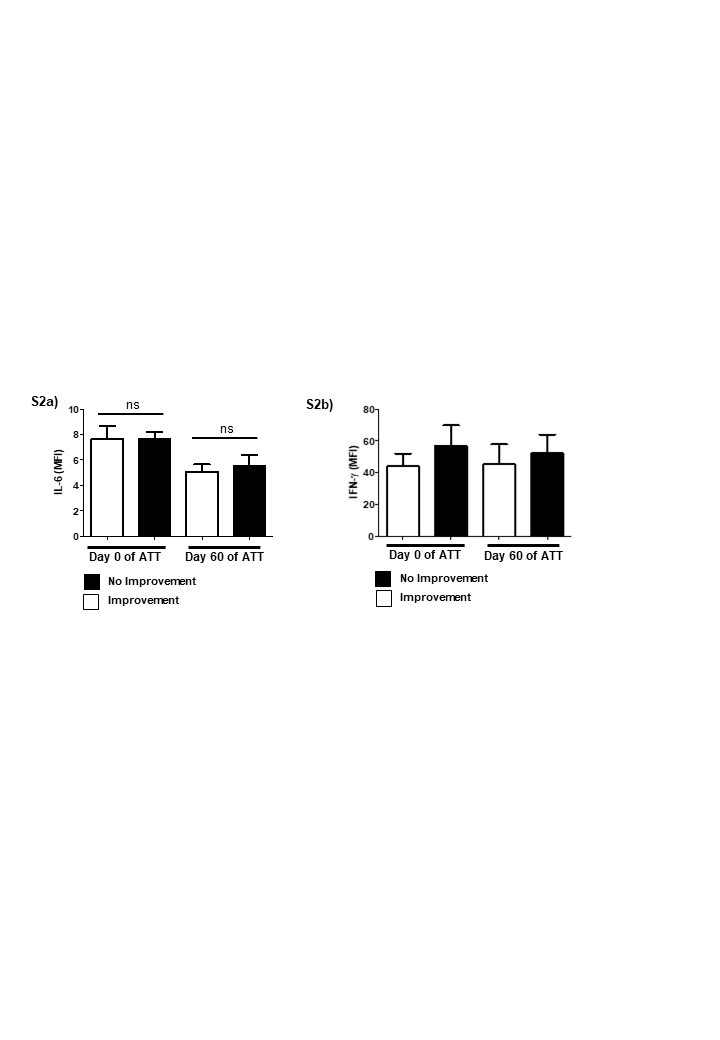

Supplement: Figure S2 — IL-6 and IFN-γ are not related with radiological improvement. (A) Comparison of IL-6 levels before and after 60 days of ATT between patients who presented radiological improvement or not after 60 days of ATT. (B) Comparison of IFN-γ levels in patients who presented or not cavity formation. Serum IL-6 and IFN-γ levels are presented as mean fluorescence intensity (MFI). Data in each figure are expressed as mean ± SD. ns–p, non-significant by 1-way ANOVA followed by Newman-Keuls test. [file Image_2.TIF]
